# 3D‐Printed Biomimetic Vascular Scaffold Crosslinked with Heparan Sulfate for Sustained Release of PDGFB‐LG4 Fusion Protein Promotes Bone Regeneration

**DOI:** 10.1002/advs.202414362

**Published:** 2025-03-27

**Authors:** Jiahua Duan, Gaofei Qian, Huikang Zhang, Feifan Wang, Qiang Tian, Dong Lei, Jianning Zhao

**Affiliations:** ^1^ Nanjing Jinling Hospital Affiliated Hospital of Medical School Nanjing University Nanjing 210023 P. R. China; ^2^ State Key Laboratory of Pharmaceutical Biotechnology School of Life Sciences Nanjing University Nanjing 210023 P. R. China; ^3^ Institute of Digital Medicine Nanjing Clinical Nuclear Medicine Centre Nanjing Medical University Nanjing 210001 P. R. China; ^4^ Department of Plastic and Reconstructive Surgery Department of Cardiology Shanghai Key Lab of Tissue Engineering Shanghai 9th People's Hospital Shanghai Jiao Tong University School of Medicine Shanghai 200011 P. R. China

**Keywords:** biomimetic vascular scaffold, bone regeneration, fusion protein, heparan sulfate, vascularization

## Abstract

A central focus of bone tissue engineering is the construction of vascular systems, which provide nutrients for cell survival, remove metabolic waste, and accelerate tissue regeneration. Platelet‐derived growth factor‐BB (PDGFB) has the ability to stimulate both vascularization and bone regeneration; however, its clinical application has been hindered by side effects and low efficacy due to suboptimal delivery systems. In this study, a biomimetic vascular scaffold crosslinked with heparan sulfate (HS) is developed to enable sustained delivery of the PDGFB‐LG4 fusion protein, targeting the regeneration of critically sized bone defects. The scaffold is designed with a vascular‐like hierarchical structure, incorporating a customized 3D framework, multibranched microchannels, and permeable porous walls, which facilitates mass exchange and cell infiltration. PDGFB‐LG4 exhibits superior osteoinductive and angiogenic activity compared to PDGFB. In a calvarial defect model, the composite scaffold (PCLHS‐PDGFB‐LG4) significantly enhances both vascularization and bone regeneration, demonstrating improved efficacy at lower doses compared to PDGFB. This approach may be applicable to other growth factors and gelatin‐based materials, offering the potential for a wide range of applications in regenerative medicine.

## Graphical abstract

Fusion protein PDGFB‐LG4 exhibits superior osteoinductive and angiogenic activity compared to PDGFB. The PCLHS‐PDGFB‐LG4 scaffold, featuring perfusable and permeable vascular‐like networks, promotes angiogenesis and osteogenesis through the sustained release of PDGFB‐LG4, thereby accelerating the bone defect repair process.

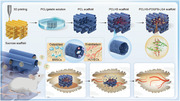

## 1 Introduction

Traumatic injury, degenerative disease, congenital defects, or the surgical removal of tumors can result in large bone defects (typically >2 cm) that do not heal spontaneously and therefore require clinical intervention.^[^ ^1^ ^]^ Bone tissue repair and regeneration are regulated by a variety of growth factors. Platelet‐derived growth factor‐BB (PDGFB), for example, is essential for granulation tissue formation following injury and for the recruitment and proliferation of stem and progenitor cells, which facilitate vascular maturation.^[^ ^2^, ^3^ ^]^ Despite its significant potential in regenerative therapies, the clinical application of growth factors has been limited.^[^ ^4^ ^]^ PDGFB delivery in clinical settings is associated with several challenges, including safety concerns and high costs.^[^ ^4^ ^]^ These issues are largely attributed to the need for supraphysiological doses of PDGFB to achieve therapeutic efficacy in humans, given the protein's short half‐life and rapid clearance in vivo.^[^ ^5^ ^]^ Localized drug delivery has emerged as a promising strategy to enhance efficacy while minimizing systemic toxicity at lower doses. Therefore, an optimized delivery system is essential for growth factor‐based tissue engineering.^[^ ^6^ ^]^

Heparan sulfate (HS), a naturally occurring glycosaminoglycan, has a strong affinity for numerous growth factors involved in fracture healing, including PDGFB.^[^ ^7^ ^]^ HS can extend the release of growth factors and protect proteins from degradation, thereby enhancing both their potency and half‐life.^[^ ^5^, ^8^ ^]^ Furthermore, HS has been shown to augment bone morphogenetic protein‐2 (BMP2)‐induced osteogenesis and fibroblast growth factor‐2 (FGF‐2)‐stimulated differentiation of mesenchymal stem cells.^[^ ^9^, ^10^ ^]^ Additionally, a growing body of evidence suggests that HS can promote MSC proliferation and improve osteogenic activity.^[^ ^10^ ^]^ However, HS‐modified scaffolds for protein delivery have not yet been reported. In this study, we explored the potential of HS as a delivery system to control PDGFB release.

The LG4 module from laminin contains major binding sites for HS and plays a critical role in early embryonic development.^[^ ^11^ ^]^ Additionally, the LG4 module promotes cell attachment through syndecans and facilitates cell spreading via integrins.^[^ ^11^ ^]^ Recently, the AG73 peptide from LG4 was added to the C‐terminus of growth factors to induce sustained, low‐intensity signaling and reduce the desensitization of growth factor receptors.^[^ ^12^ ^]^ This fusion protein retains the functions of both PDGFB and LG4. We hypothesized that PDGFB‐LG4, with its enhanced affinity for HS, could provide a more controlled and sustained release from HS, potentially resulting in superior morphogenetic activity and improved tissue regeneration.

Numerous biomaterial‐based delivery systems have been developed to efficiently deliver growth factors.^[^ ^13^ ^]^ These include hydrogels, nanoparticles, and 3D‐printed scaffolds designed to sustain growth factor release and promote tissue repair.^[^ ^14^, ^15^, ^16^ ^]^ Efficient delivery systems must mimic the in vivo slow release and controlled signaling of growth factors. Among these delivery vehicles, 3D‐printed scaffolds have garnered increased attention for bone tissue regeneration due to their customizable porous architecture.^[^ ^17^ ^]^ Our previous studies demonstrated that the microchannel structures of poly(e‐caprolactone) (PCL)‐based 3D‐printed scaffolds not only enhance oxygen diffusion and nutrient transport but also facilitate tissue revascularization and integration.^[^ ^17^, ^18^ ^]^ However, since PCL is an inert biomaterial that lacks active functional groups, achieving the sustained release of active biological factors necessary for guiding angiogenesis and osteogenesis remains difficult. Therefore, fabricating a versatile 3D‐printed scaffold that possesses both a vascular‐like structure and a localized drug delivery system remains a significant challenge.

In this study, we engineered a biomimetic vascular scaffold crosslinked with HS for the sustained delivery of PDGFB variants to promote the regeneration of critically sized bone defects. To replicate the perfusable and permeable channel structures of natural microvascular networks, we utilized 3D‐printed sacrificial caramel templates and a poly(e‐caprolactone) (PCL)/gelatin composite polymer coating with integrated phase separation to create the PCL/gelatin scaffold (referred to as the PCL scaffold in this study). HS was crosslinked with gelatin to form the PCLHS scaffold, and PDGFB‐LG4 was immobilized on the PCLHS scaffold through electrostatic interactions to construct the bioactive PCLHS‐PDGFB‐LG4 scaffold. We hypothesized that these angiogenic biomaterials could localize the release of PDGFB variants within the defect site, thereby reducing the required dose of growth factors and resulting in enhanced bone tissue regeneration.

## 2 Results

### 2.1 Fabrication and Characterization of Biomimetic Vascular Scaffolds

**Figure 1:**
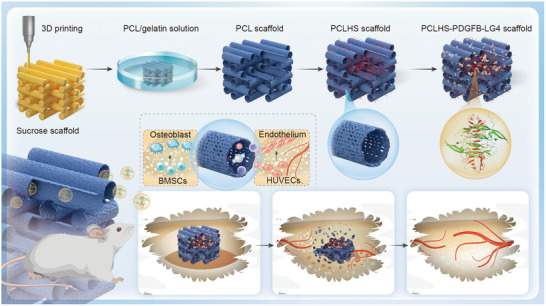
Schematic illustration of the preparation of the PCLHS‐PDGFB‐LG4 scaffold. The PCLHS‐PDGFB‐LG4 scaffold, featuring perfusable and permeable vascular‐like networks, promotes angiogenesis and osteogenesis through the sustained release of PDGFB‐LG4, thereby accelerating the bone defect repair process.

**Figure 2:**
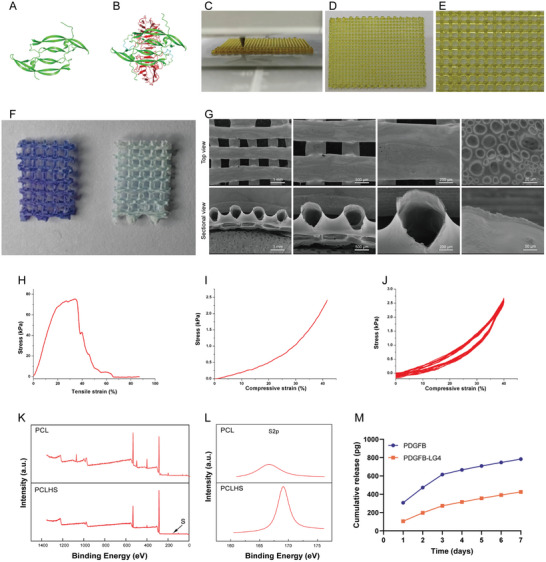
Characterization of the biomimetic scaffold. A) Predicted structure of PDGFB. B) Predicted structure of PDGFB‐LG4. C) 3D printing of caramel ink. D,E) 3D‐printed sacrificial template. F) Toluidine blue staining of the scaffolds. Left: PCLHS scaffold; Right: PCL scaffold. G) SEM top view and sectional view of the PCL scaffold. H–J) Mechanical strain‐stress curves of PCL scaffold: H) Tensile fracture test, I) Uniaxial compressive test, J) Cyclic compression test. K, L) XPS analysis of PCL and PCLHS scaffolds, M) Release profile of recombinant protein from the PCLHS scaffold (n = 3 per group).

A reverse‐molding 3D‐printing method was employed to fabricate a biomimetic vascular scaffold with a vascular‐like hierarchical structure (**Figure** **1**). Sucrose was gradually caramelized through heating, which gave it excellent extrusion formability, allowing it to be extruded into continuous single fibers using a 3D printer (**Figure** **2**C). Multilayered templates were easily created by stacking the melted caramel ink in 3D according to the printing path (Figure 2D,E). The 3D printing parameters directly influenced both the macroscopic and microscopic structures of the template, significantly impacting the morphology of the resulting biomimetic vascular network scaffolds.

Scanning electron microscopy (SEM) revealed that the scaffolds exhibited a multi‐level vascular‐like structure (Figure 2G). The microchannels were stacked to form a multilayer hollow framework, with the channels interconnected to create a multibranch network. Numerous micropores were present on the surface of the channels, enabling permeability through the channel walls. This vascular‐like structure could support physiologically relevant perfusion, mimicking a microvascular network for mass transfer in 3D.

Despite the thin and porous channel walls, the scaffolds demonstrated excellent ductility, with a tensile modulus of 477.5 ± 99.6 kPa, tensile strength of 87.8 ± 19.8 kPa, and elongation at break of 25.6 ± 8.9% (Figure 2H). Uniaxial compression tests indicated that the scaffolds had a modulus of 13.4 ± 1.7 kPa (Figure 2I). Additionally, cyclic compression tests demonstrated superior elasticity and anti‐fatigue properties, with minimal hysteresis under dynamic pressure (Figure 2J). Moreover, the elasticity of the aligned channel architecture enabled the scaffolds to resist multiple tensile and compression deformations, allowing them to maintain their original tubular structure during various applications.

### 2.2 Conjugation of Heparan Sulfate to Biomimetic Vascular Scaffolds and Protein Release

We performed X‐ray photoelectron spectroscopy (XPS) to confirm HS coating on PCL scaffold surfaces. The XPS result showed that the PCLHS scaffold had one peak at 168.86 eV, corresponding to the S atoms present on the PCLHS scaffold surface (Figure 2K,L). No such peak was observed in the S2p peaks for the PCL scaffold. To further prove whether the HS was successfully crosslinked to the scaffold, we performed the toluidine blue staining of scaffolds (Figure 2F). It was known that toluidine blue staining could bind HS through electrostatic adsorption. The PCL scaffold showed no blue staining, while the PCLHS scaffold showed exhibited blue staining throughout the scaffold. Overall, both XPS and toluidine blue staining confirm successful HS conjugation on the scaffold surface. The structure of PDGFB and PDGFB‐LG4 was predicted by Alphfold3 (Figure 2A,B). In the protein release experiment, we studied the release behavior of PDGFB and PDGFB‐LG4 on the PCLHS scaffold. The results showed that the release rate of PDGFB was faster, while the release rate of PDGFB‐LG4 was relatively slower. This indicates that the HS has stronger adsorption for PDGFB‐LG4, showing high affinity, thus delaying the release of PDGFB‐LG4 (Figure 2M).

### 2.3 In Vitro Cell Proliferation and Attachment Assay of HS, PDGFB, and PDGFB‐LG4

**Figure 3:**
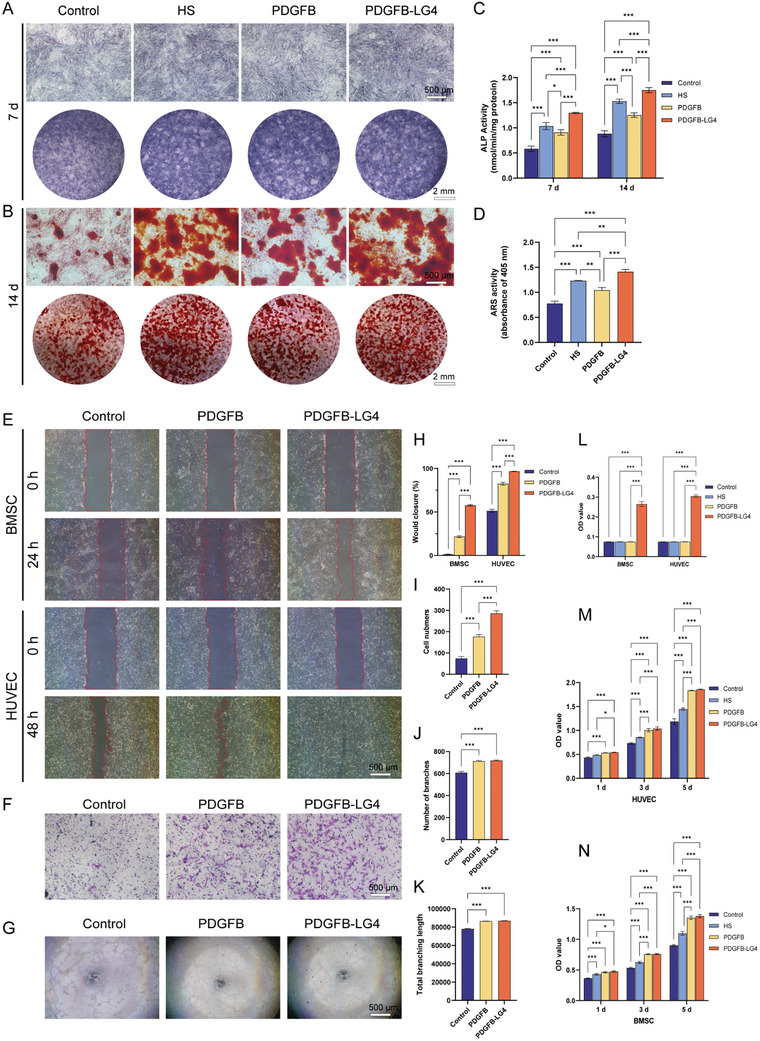
Evaluation of the osteogenic and angiogenic potential of HS and PDGFB variants. A) Representative images of ALP staining of BMSCs treated with HS or PDGFB variants on day 7 (n = 3 per group). B) Representative images of Alizarin Red S staining of BMSCs treated with HS or PDGFB variants on day 14 (n = 3 per group). C) Quantitative measurement of ALP activity on days 7 and 14 across different groups (n = 3 per group). D) Quantitative measurement of Alizarin Red S staining on day 14 across different groups (n = 3 per group). E) Representative images of the scratch wound healing assay (n = 3 per group). F) Representative images of the transwell migration assay (n = 3 per group). G) Tube formation assay of HUVECs (n = 3 per group). H) Percentage of wound closure determined by microscopic photographs (n = 3 per group). I) Quantitative measurement of cell numbers in the transwell migration assay (n = 3 per group). J, K) Quantitative measurement of angiogenic parameters in the tube formation assay (n = 3 per group). L) Quantitative measurement of the cell attachment assay (n = 3 per group). M,N) CCK‐8 results for BMSCs and HUVECs treated with HS or PDGFB variants (n = 3 per group). Statistical analysis was performed using one‐way or two‐way analysis of variance (ANOVA) with Tukey's multiple comparisons test. A *p*‐value of less than 0.05 was considered statistically significant: ^*^ *p* < 0.05, ^**^ *p* < 0.002, and ^***^ *p* < 0.001.

To evaluate cell proliferation, a CCK‐8 assay was performed using human umbilical vein endothelial cells (HUVECs) and bone marrow mesenchymal stem cells (BMSCs). Compared with the control group, the HS, PDGFB, and PDGFB‐LG4 groups significantly promoted cell proliferation at 1, 3, and 5 days (**Figure** **3**M,N). HS, PDGFB, and PDGFB‐LG4 were also analyzed for their effects on cell attachment using HUVECs and BMSCs. Notably, only PDGFB‐LG4 enhanced cell attachment in both HUVECs and BMSCs (Figure 3L), while neither HS nor PDGFB showed any improvement in cell attachment.

### 2.4 Analysis of HS, PDGFB, and PDGFB‐LG4 on Osteogenic Activity

The osteogenic effects of HS, PDGFB, and PDGFB‐LG4 were evaluated through both quantitative and qualitative analyses of alkaline phosphatase (ALP) activity, calcium deposition, and osteogenic gene expression. ALP staining of BMSCs cultured with HS, PDGFB, or PDGFB‐LG4 is shown in Figure 3A. Compared with the control group, the HS, PDGFB, and PDGFB‐LG4 groups significantly enhanced ALP activity in BMSCs at days 7 and 14 (Figure 3C). Notably, the PDGFB‐LG4 group demonstrated a significantly higher increase in ALP activity compared to the PDGFB group and exhibited the highest levels among all groups.

To assess mineral deposition in BMSCs treated with HS, PDGFB, and PDGFB‐LG4, Alizarin Red S staining was performed at 14 days. Increased mineral matrix formation was observed and quantified in the HS, PDGFB, and PDGFB‐LG4 groups, with the PDGFB‐LG4 group showing the most pronounced effect (Figure 3B). Compared with the PDGFB group, the PDGFB‐LG4 group produced a greater amount of mineral matrix. Quantitative measurements of Alizarin Red S staining are presented in Figure 3D.

**Figure 4:**
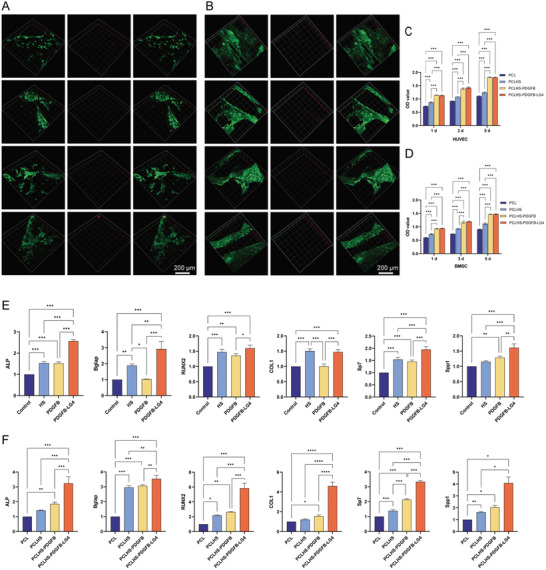
Biocompatibility and in vitro osteogenesis of the composite scaffolds. A,B) Live‐dead staining of BMSCs and HUVECs on different composite scaffolds (n = 3 per group). C,D) CCK‐8 results of BMSCs and HUVECs on different composite scaffolds (n = 3 per group). E) Osteogenesis‐related gene expression of BMSCs treated with HS or PDGFB variants (n = 3 per group). F) Osteogenesis‐related gene expression of BMSCs cultured on different composite scaffolds (n = 3 per group). Statistical analysis was performed using ANOVA with Tukey's multiple comparisons test. A *p*‐value of less than 0.05 was considered statistically significant: ^*^ *p* < 0.05, ^**^ *p* < 0.002, and ^***^ *p* < 0.001.

Quantitative real‐time PCR (qRT‐PCR) analysis revealed that both HS and PDGFB‐LG4 significantly upregulated the expression of osteogenesis‐related genes, including alkaline phosphatase (ALP), secreted phosphoprotein 1 (Spp1), runt‐related transcription factor 2 (RUNX2), type I collagen (COL1), osterix (Sp7), and bone gamma‐carboxyglutamate protein (Bglap) (**Figure** **4**E). In conclusion, these results suggest that HS and PDGFB‐LG4 significantly promote the osteogenic differentiation of BMSCs.

### 2.5 Analysis of PDGFB and PDGFB‐LG4 on Angiogenic Activities

Cell migration is pivotal to the wound‐healing, especially of the HUVECs and BMSCs, which can induce angiogenesis at the injury site and promote bone regeneration.^[^ ^19^ ^]^ Scratch wound healing assay showed that the cell migration of both HUVECs and BMSCs in the PDGFB and PDGFB‐LG4 group was much better than that of the control group (Figure 3E). However, PDGFB‐LG4 promotes cell migration of both HUVECs and BMSCs significantly better than PDGFB. The quantitative analysis further indicated the best wound closure in the PDGFB‐LG4 group at 24 h of BMSCs and 48 h of HUVECs (Figure 3H). Besides, the potential of PDGFB variants to induce the HUVECs migration was further validated by an in vitro transwell migration assay. Both PDGFB and PDGFB‐LG4 groups showed an increased number of HUVECs migration to lower chambers than the control group (Figure 3F). Importantly, better HUVECs migration was observed and quantitatively measured in the PDGFB‐LG4 group than PDGFB group (Figure 3I). Taken together, these results suggested that the PDGFB‐LG4 showed a significantly promotive effect on cell migration. The blood vessel plays an important role in the process of bone reconstruction. To ascertain the angiogenic potential of PDGFB variants, in vitro tube formation evaluation was performed using HUVECs. It was clearly seen that PDGFB had PDGFB‐LG4 had significantly better tubular network formation as compared to the control group (Figure 3G). Furthermore, the quantitative parameters, including the number of branches, and total branch length, were profoundly better in the PDGFB and PDGFB‐LG4 groups than those of the control group (Figure 3K). However, there was no significant difference between the PDGFB group and the PDGFB‐LG4 group in terms of the quantitative tubular network parameters.

### 2.6 Biocompatibility and Osteogenesis Evaluation of the Composite Scaffolds

To evaluate the biocompatibility of the composite scaffolds, BMSCs or HUVECs were seeded onto PCL scaffolds (PCL), PCLHS scaffolds (PCLHS), PCLHS scaffolds with PDGFB (PCLHS‐PDGFB), and PCLHS scaffolds with PDGFB‐LG4 (PCLHS‐PDGFB‐LG4). The CCK‐8 assay results showed that the number of cells in all groups increased over 5 days (Figure 4C,D). Encouragingly, both the PCLHS‐PDGFB and PCLHS‐PDGFB‐LG4 groups significantly promoted cell proliferation compared to the other groups. PCLHS moderately enhanced cell proliferation relative to the PCL group. Live‐dead staining revealed that BMSCs and HUVECs adhered and spread efficiently across all groups, demonstrating good biocompatibility (Figure 4A,B). Notably, cells adhered to the microfilaments after 5 days of culture.

The effect of the composite scaffolds on osteogenesis‐related gene expression in BMSCs after 14 days of incubation was confirmed by qRT‐PCR analysis (Figure 4F). The PCLHS‐PDGFB‐LG4 group exhibited the highest expression levels of osteogenesis‐related genes among all groups.

### 2.7 Osteogenic Mechanism of the Composite Scaffolds

**Figure 5:**
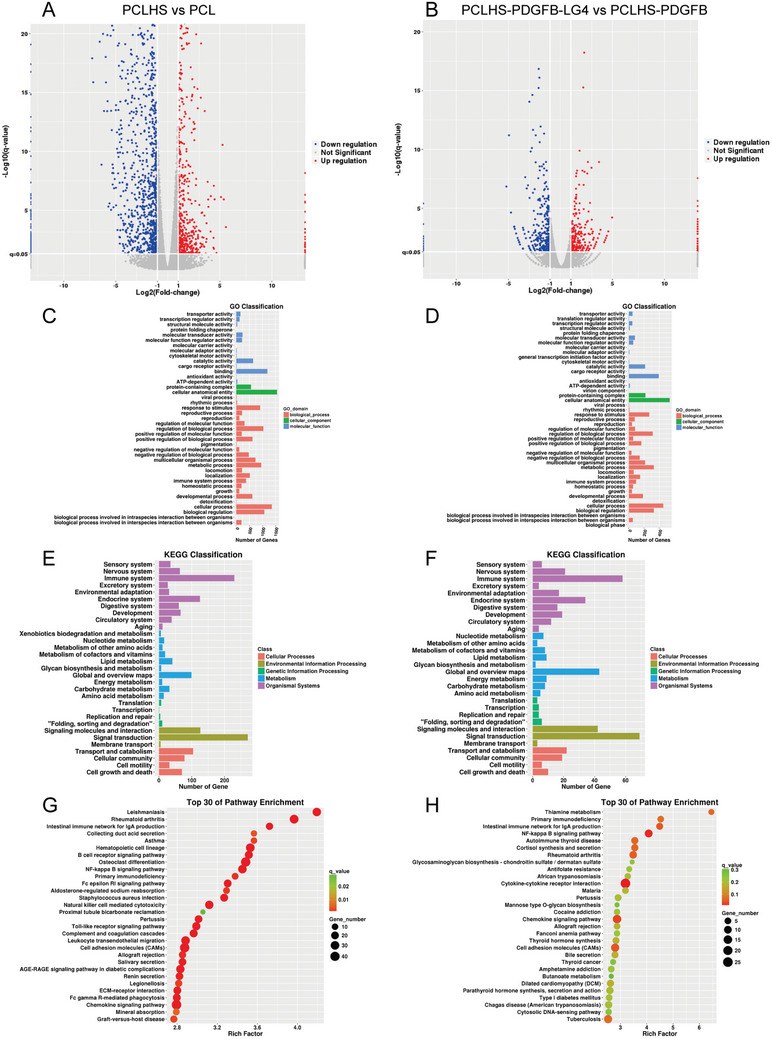
RNA‐seq results of composite scaffolds. A,C,E,G) Volcano plots, GO classification, KEGG classification, and KEGG enrichment comparison between PCLHS scaffold and PCL scaffold (n = 2 per group). B,D,F,H) Volcano plots, GO classification, and KEGG classification, and KEGG enrichment comparison between PCLHS‐PDGFB‐LG4 scaffold and PCLHS‐PDGFB scaffold (n = 2 per group).

To further investigate the potential mechanisms underlying the effects of the composite scaffolds on BMSCs, RNA sequencing was performed on the PCL, PCLHS, PCLHS‐PDGFB, and PCLHS‐PDGFB‐LG4 groups (**Figure** **5**; Figure S2, Supporting Information). The volcano plot revealed that the PCLHS group exhibited distinct gene expression profiles compared to the PCL group, suggesting that HS significantly influenced BMSCs on the scaffold. Furthermore, the volcano plot indicated that the PCLHS‐PDGFB‐LG4 group had distinct gene expression patterns compared to the PCLHS‐PDGFB group, demonstrating that PDGFB‐LG4 further modulated the behavior of the seeded BMSCs. To elucidate the functions of highly expressed genes in the different scaffold groups, gene ontology (GO) analysis was conducted. The GO analysis displayed differential gene expression between the PCL and PCLHS groups, as well as between the PCLHS‐PDGFB and PCLHS‐PDGFB‐LG4 groups. Similarly, the Kyoto Encyclopedia of Genes and Genomes (KEGG) analysis revealed clear differences in gene expression across these groups. KEGG pathway enrichment analysis showed that Toll‐like receptor (TLR) signaling pathways were activated in the PCLHS group compared to the PCL group. Additionally, NF‐kappa B signaling pathways and cell adhesion molecules were activated in the PCLHS‐PDGFB‐LG4 group compared to the PCLHS‐PDGFB group.

### 2.8 Promotion of In Vivo Bone Formation by the Composite Scaffolds

**Figure 6:**
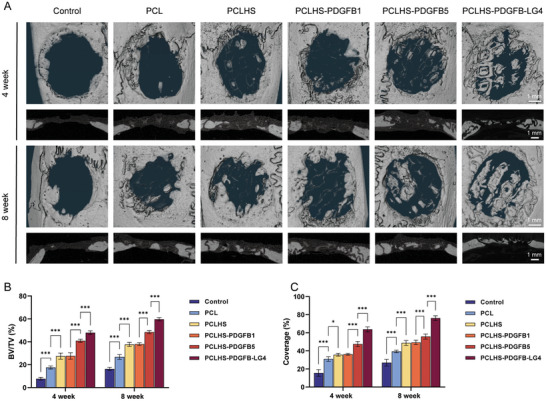
In vivo bone regeneration assessment of different scaffolds. A) Representative 3D‐reconstructed micro‐CT images and sectional view images of the defect sites after scaffold implantation for 4 and 8 weeks (n = 6 per group). B) BV/TV analysis determined by micro‐CT at 4 and 8 weeks (n = 6 per group). C) Coverage analysis determined by micro‐CT at 4 and 8 weeks (n = 6 per group). Statistical analysis was performed using ANOVA with Tukey's multiple comparisons test. A p‐value of less than 0.05 was considered statistically significant: ^*^ *p* < 0.05, ^**^ *p* < 0.002, and ^***^ *p* < 0.001.

We started to assess the in vivo efficacy of the scaffolds combined with PDGFB variants in a calvarial defect model in rats. In this model, bone defects of 5 mm in diameter were surgically created and treated with empty control (Control), PCL scaffolds (PCL), PCLHS scaffolds (PCLHS), PCLHS scaffolds with 1 µg PDGFB (PCLHS‐PDGFB1), PCLHS scaffolds with 5 µg PDGFB (PCLHS‐PDGFB5), and PCLHS scaffolds with 1 µg PDGFB‐LG4 (PCLHS‐PDGFB‐LG4). Composite scaffolds were implanted into the rat calvarial defect for 4 weeks and 8 weeks to observe bone formation. After scaffold implantation at 4 and 8 weeks, the specimens were collected for micro‐CT, histological staining, and immunofluorescence analysis. Representative 3D‐reconstructed micro‐CT images of the defect sites are presented in **Figure** **6**A. At 4 weeks, all scaffold groups showed more newly formed bone compared to the control group, with the PCLHS‐PDGFB‐LG4 group displaying the best bone regeneration. New bone formation was clearly visible covering the scaffold surface and microfilaments in all scaffold groups, and new bone grew along the scaffold microfilaments. Compared with the PCL group, the PCLHS group generated more new bone. We observed that both defect coverage and bone formation increased with the dose of PDGFB. The PCLHS‐PDGFB5 group generated significantly more new bone than the PCLHS‐PDGFB1 group, while the PCLHS‐PDGFB1 group showed nearly the same new bone formation as the PCLHS group, with no significant difference. By 8 weeks post‐implantation, more newly formed bone was observed in all groups, with the PCLHS‐PDGFB‐LG4 group again demonstrating the best bone regeneration. In this group, new bone growth was evident in the central area of the defects, with bone tissue forming in the peripheral area near the original bone as well. BV/TV and coverage analysis are presented in Figure 6B,C.

**Figure 7:**
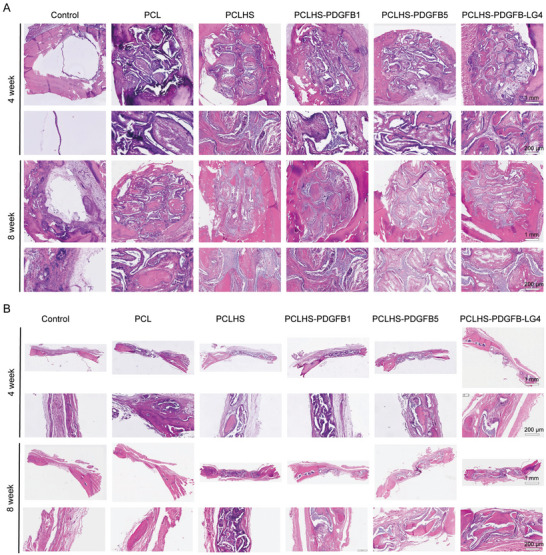
Evaluation of in vivo bone regeneration through histological staining. A) HE staining of the top view of bone defects in rats at 4 and 8 weeks (n = 6 per group). B) HE staining of the sectional view of bone defects in rats at 4 and 8 weeks (n = 6 per group).

**Figure 8:**
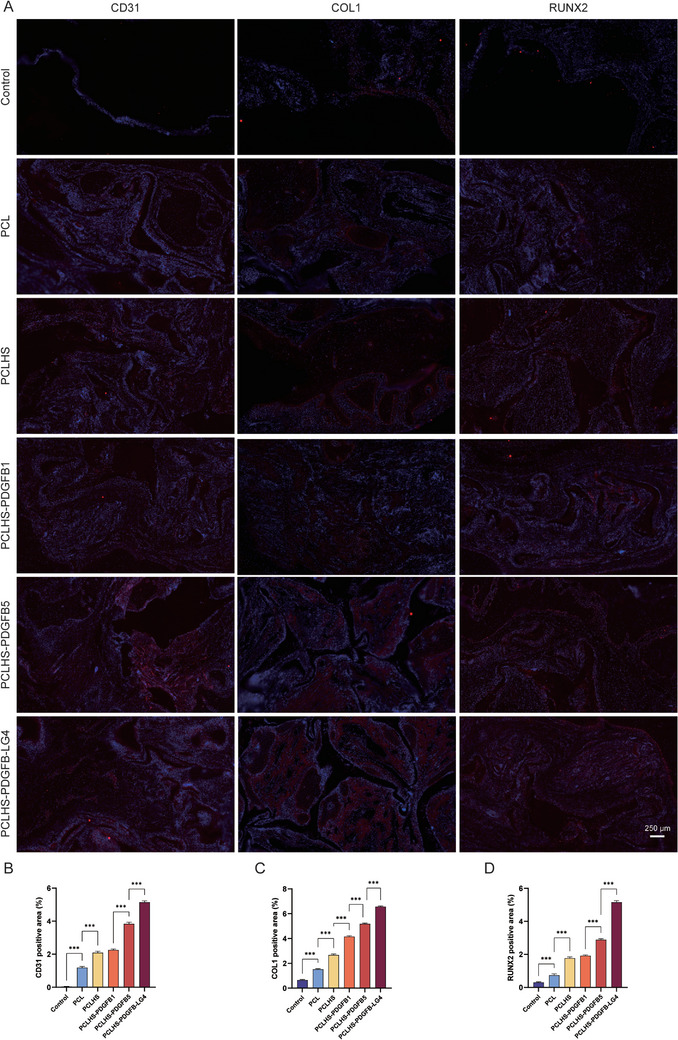
Evaluation of in vivo bone regeneration through Immunofluorescence staining. A) Representative images of immunofluorescence staining of CD31, COL1, and RUNX2 expression at 8 weeks after scaffold implantation, with cell nuclei were stained with DAPI (n = 3 per group). B–D) Quantitative analysis of CD31, COL1, and RUNX2 positive areas using ImageJ software (n = 3 per group). Statistical analysis was performed using ANOVA with Tukey's multiple comparisons test. A *p*‐value of less than 0.05 was considered statistically significant: ^*^ *p* < 0.05, ^**^ *p* < 0.002, and ^***^ *p* < 0.001.

The scaffold‐mediated bone regeneration was further verified by histological analysis, including hematoxylin and eosin (HE) and Masson's trichrome staining (**Figure** **7**A,B; Figure S3, Supporting Information). Observations of HE staining images at 4 and 8 weeks post‐implantation showed no obvious inflammatory response or necrosis in any group, indicating good biocompatibility of the scaffolds. In the control group, only minimal new bone was observed at the edge of the defect region. In contrast, new bone formation extended into the scaffold microfilaments in the other groups, with the PCLHS‐PDGFB‐LG4 group showing the most pronounced effect. Importantly, new blood vessels were observed in the defect area across all scaffold groups. The PCLHS‐PDGFB‐LG4 and PCLHS‐PDGFB5 groups exhibited more vascularization than the other groups. Masson's trichrome staining further confirmed that the PCLHS‐PDGFB‐LG4 group exhibited new bone tissue formation from the edge to the central area of the defect. Consistent with the bone formation results, the PCLHS‐PDGFB1 group showed a similar level of new blood vessel formation to the PCLHS group. To further confirm the in vivo vascularization and bone regeneration effects of the composite scaffolds, immunofluorescence staining for CD31, COL1, and RUNX2 was performed at 8 weeks (**Figure** **8**). The PCLHS‐PDGFB‐LG4 group displayed the highest fluorescence signal for these markers among all groups, consistent with the previous results.

## 3 Discussion

A major focus of tissue engineering is the construction of vascular systems after implantation, which provide nutrients for growing cells, remove metabolic waste, and accelerate tissue regeneration. Previous studies have shown that microchannel structures promote angiogenesis by inducing the tubular formation of endothelial cells, which align along the channel surface to form an endothelium.^[^ ^20^ ^]^ However, scaffolds lacking bioactive factors often result in insufficient bone regeneration.^[^ ^12^, ^16^, ^21^ ^]^ Growth factors are essential for tissue regeneration and hold great promise in regenerative medicine, yet many growth factors that have entered clinical trials either failed or raised significant safety concerns. Issues with safety and cost‐effectiveness are often linked to suboptimal delivery systems, which can lead to unsatisfactory results.^[^ ^22^ ^]^ Therefore, there is a pressing need to develop more efficient systems to optimize growth factor delivery and signaling. In this study, we present a biomimetic vascular‐like scaffold crosslinked with HS for the sustained delivery of PDGFB‐LG4, aimed at promoting bone regeneration and vascularization. Our results demonstrate that HS is an excellent candidate for sustaining growth factor release and that PDGFB‐LG4 exhibits superior osteoinductive and angiogenic activity.

Heparin‐mediated delivery systems for growth factors have recently been reported to achieve sustained release while maintaining the bioactivity of heparin‐binding growth factors.^[^ ^5^, ^8^ ^]^ However, heparin inhibits cell growth and has anticoagulant effects, which may hinder tissue regeneration.^[^ ^8^, ^23^ ^]^ HS is a highly sulfated linear polysaccharide composed of repeated disaccharide units typically attached to a core protein.^[^ ^12^ ^]^ Sulfation along the HS polysaccharide chain forms short protein‐binding domains, separated by longer unsulfated regions.^[^ ^24^ ^]^ Compared with heparin, HS offers better biocompatibility. The binding of growth factors to HS protects them from proteolytic degradation and prolongs their half‐life.^[^ ^25^ ^]^ Moreover, HS binding is thought to be crucial in providing a matrix‐bound or cell‐surface‐bound reservoir of growth factors, promoting long‐term availability and enhancing their function.^[^ ^12^ ^]^ In this study, we demonstrated that HS promotes the proliferation of BMSCs and enhances osteogenic activity in vitro. In the rat calvarial bone defect model, the PCLHS group exhibited improved bone regeneration compared to the PCL group. To further explore the mechanism underlying the osteogenic effects of the PCLHS scaffold, we investigated the gene expression profiles of the PCLHS and PCL groups using RNA sequencing. Kyoto Encyclopedia of Genes and Genomes (KEGG) pathway enrichment analysis revealed that Toll‐like receptor (TLR) signaling pathways were activated in the PCLHS group. Previous studies have shown that TLR pathway activation promotes the osteogenic differentiation of BMSCs.^[^ ^26^ ^]^ Thus, HS serves as an excellent candidate for sustained growth factor release.

From a cost‐effectiveness and regulatory perspective, regenerative strategies involving multiple recombinant proteins are particularly challenging and significantly increase the risk of adverse effects, which is a major concern with growth factor‐based therapeutics.^[^ ^27^, ^28^ ^]^ Likely due to these issues, no regenerative therapies using multiple growth factors have been approved to date. However, even delivery systems that release a single growth factor often yield unsatisfactory regenerative outcomes.^[^ ^12^ ^]^ PDGFB not only stimulates bone regeneration but also supports angiogenesis by recruiting pericytes and HUVECs.^[^ ^29^ ^]^ While PDGFB is approved as an alternative to bone autograft during ankle and hindfoot fusion surgeries,^[^ ^30^ ^]^ some studies have failed to demonstrate significant effects.^[^ ^12^, ^31^ ^]^ Improving the efficacy of PDGFB at lower doses while ensuring safety remains an ongoing goal. We aimed to enhance the regenerative activity of PDGFB by utilizing a fusion protein. Laminins, the most abundant extracellular matrix glycoproteins, are involved in a variety of biological processes, including angiogenesis, neural development, skin reepithelialization, and wound healing.^[^ ^32^, ^33^, ^34^, ^35^ ^]^ Laminins provide substrates for cells and regulate cellular adhesion, proliferation, migration, and differentiation.^[^ ^36^ ^]^ Importantly, the expression of LG4 domains from laminin is upregulated in wounds after injury.^[^ ^37^, ^38^ ^]^ The LG4 module from laminin has a high affinity for HS, which can be leveraged for controlled growth factor release. Through protein engineering, we created a recombinant protein, PDGFB‐LG4, by fusing LG4 to PDGFB. PDGFB‐LG4 promotes the proliferation and attachment of both BMSCs and HUVECs, as LG4 enhances cell attachment via syndecans.^[^ ^11^ ^]^ Previous studies have demonstrated that LG4 promotes cell spreading through integrins, leading to enhanced osteogenic differentiation of BMSCs.^[^ ^11^, ^39^, ^40^ ^]^ We then tested whether PDGFB‐LG4 could improve bone regeneration. As expected, PDGFB‐LG4 exhibited significantly better osteogenic activity compared to PDGFB in vitro. The LG4 module has also been shown to promote blood vessel formation and cell migration via syndecan binding.^[^ ^41^, ^42^ ^]^ Compared with PDGFB, PDGFB‐LG4 demonstrated superior angiogenic activity in HUVECs. Owing to LG4's high affinity for HS, PDGFB‐LG4 exhibited a slower release from the PCLHS scaffold compared to PDGFB. This strong local retention of PDGFB‐LG4 to the scaffold through electrostatic interactions enhances on‐site therapeutic efficacy while minimizing side effects in neighboring and distant tissues.^[^ ^43^ ^]^

In the calvarial bone defect model, the PCLHS‐PDGFB‐LG4 group exhibited the most effective bone regeneration and vascularization among all groups. To further elucidate the underlying mechanism of PCLHS‐PDGFB‐LG4's osteogenic induction, we investigated the gene expression profile of BMSCs through RNA sequencing. Compared with the PCLHS‐PDGFB group, KEGG signaling pathway enrichment analysis revealed that the NF‐kappa B signaling pathway and cell adhesion molecules were activated in the PCLHS‐PDGFB‐LG4 group. Previous studies have shown that activation of the NF‐κB pathway enhances the osteogenic differentiation of BMSCs.^[^ ^44^ ^]^ Consistent with studies demonstrating that LG4 promotes cell attachment and spreading, RNA sequencing indicated that cell adhesion molecules were activated in the PCLHS‐PDGFB‐LG4 group, contributing to enhanced osteogenic differentiation of BMSCs.^[^ ^45^, ^46^ ^]^ We also demonstrated that PDGFB‐LG4 improved bone regeneration at lower doses compared to PDGFB. Specifically, 1 µg of PDGFB‐LG4 exhibited significantly better bone regeneration and vascularization than 5 µg of PDGFB. In contrast, 1 µg of PDGFB had no detectable effect on bone regeneration. Thus, PDGFB‐LG4 reduces the therapeutic dose of growth factors, which is expected to lower the risk of side effects.^[^ ^47^ ^]^

Natural bone is highly vascularized and relies on blood vessels for the timely supply of oxygen and nutrients to maintain skeletal integrity.^[^ ^48^ ^]^ One of the major challenges in tissue regeneration is providing suitable vascularization to 3D scaffolds, as oxygen diffusion is limited to a distance of 150 to 200 µm from the nearest blood vessels.^[^ ^49^ ^]^ The success of 3D‐constructed tissues, therefore, heavily depends on angiogenesis, which has been shown to promote osteogenesis.^[^ ^50^ ^]^ However, current bone tissue engineering strategies often fail to produce new bone with a sufficient density of blood vessels. Several approaches have been proposed to enhance vascularization, including the design of biomimetic 3D scaffolds, the delivery of angiogenic growth factors like vascular endothelial growth factor (VEGF), and the use of potent cell sources such as stem cells or mature vascular cells.^[^ ^51^, ^52^, ^53^ ^]^ Despite these efforts, increasing vascularization in new bone has met with limited success. Angiogenesis is known to rely on both cell–cell and cell‐matrix adhesive interactions.^[^ ^54^, ^55^ ^]^ The vascular like hierarchical structure, multibranched microchannels, and permeable porous walls, which facilitated mass exchange and cell infiltration, combined with the superior angiogenic activity of PDGFB‐LG4, led to the PCLHS‐PDGFB‐LG4 scaffold exhibiting the best vascularization performance.

While the results of the animal studies demonstrate the potential of the PCLHS‐PDGFB‐LG4 scaffold for bone regeneration, several challenges remain in transitioning this technology to clinical practice. These include the immunogenicity of the composite scaffold, which needs to be thoroughly evaluated in human trials, and the production costs, which could limit large‐scale manufacturing. Additionally, efforts should be made to optimize the scalability of the scaffold production process to ensure consistent quality and cost‐effectiveness. Future studies should focus on addressing these challenges, particularly through the use of cost‐efficient manufacturing techniques.

## 4 Conclusion

In this study, we successfully engineered a biomimetic vascular scaffold crosslinked with HS for the sustained delivery of the PDGFB‐LG4 fusion protein, promoting bone regeneration at lower doses. The vascular‐like scaffold featured multibranched microchannels and permeable porous walls, facilitating mass exchange and cell infiltration. PDGFB‐LG4 demonstrated superior osteoinductive and angiogenic activity compared to PDGFB. We also demonstrated that HS is an excellent candidate for sustained growth factor release. This approach may be applicable to other growth factors and gelatin‐based materials, offering the potential for a wide range of applications in regenerative medicine.

## 5 Experimental Section

### Fabrication and Characterization of Biomimetic Vascular Scaffold

The caramel‐based templates were 3D‐printed as previously reported.^[^ ^17^ ^]^ Sucrose was pre‐heated at 160 °C in heating storage for 30 min. The caramel ink was printed into three‐layered constructs at 130–135 °C. The nozzle size was 0.8 mm. The center‐to‐center distance between the filaments was 1.5 mm with 0°/90° lay‐down patterns between two successive layers. The layer height was 0.45 mm. For fabrication of scaffolds, PCL (Mn 50 000 g mol^−1^, Sigma–Aldrich) and gelatin (>99%, Sigma–Aldrich) were dissolved in hexafluoroisopropanol (analytical grade, >99%, Sigma–Aldrich) (4%, wt/vol) at a 9:1 weight ratio. The caramel‐based template was immersed in the mixture solution and then suspended in the air environment for 30 min to remove the solvent. PCL/Gelatin coating was formed on the surface of the template, and the phase separation process formed a large number of micropores on the surface of the coating. The coated template was placed into glutaraldehyde–water–ethanol solution (10%:30%:60%, vol/vol/vol) for 5 min to cure the gelatin. Then, the PCL/Gelatin scaffolds were soaked in distilled water to remove the caramel‐based template. Finally, the PCL/Gelatin scaffolds were freeze‐dried in a vacuum. PCL/Gelatin scaffolds were sputtered with gold and then observed in top view and sectional view to illustrate the multi‐level structure at different magnifications using Scanning Electron Microscopy (Hitachi, SU8010) with an accelerating voltage of 5 kV. The mechanical properties of PCL/Gelatin scaffolds were evaluated by tensile test and compression test using the mechanical testing machine (Instron‐5542) in a dry state. Scaffolds were cut into strips and stretched along the channel direction in simple tensile tests at a rate of 10 mm min^−1^. In the axial compression test, the scaffolds were compressed to the maximal strain of 40% at a rate of 10 mm min^−1^. In the cyclic compression test, the specimens were compressed to a large strain of 40% for 10 cycles at a rate of 10 mm min^−1^.

### Expression of Recombinant Proteins

To design the fusion protein PDGFB‐LG4, LG4 was added at the C‐terminus of PDGF‐BB, as PDGFB dimerization was important for its binding function. Amino acid sequences are provided in Table S1 (Supporting Information). For functional studies, proteins were expressed in Expi293F cells. All proteins were expressed at 37 °C in a humidified environment containing 5% CO_2_. Cell culture supernatants were harvested 3–4 days post‐transfection and individual proteins and were purified from the supernatants using Ni‐NTA affinity chromatography in 20 mm Tris‐Hcl pH 7.4 with 150 mm NaCl with 20 mm imidazole (for washing) and 250 mm imidazole (for elution). The purification yields of PDGFB and PDGFB‐LG4 extracted from the culture supernatant were 4.3 and 14.5 mg L^−1^, respectively. For subsequent cell and animal experiments, the protein buffer was replaced with PBS buffer and over 0.22 µm filter membrane to remove bacteria.

### Conjugation of Heparan Sulfate on Biomimetic Vascular Scaffolds and Protein Immobilization

Briefly, the reactive amine groups (─NH_2_) on the PCL/gelatin scaffolds were conjugated with the carboxyl groups (─COOH) on the heparan sulfate molecules, forming an amide bond (covalent bond). Heparan sulfate salt (Aladdin, Cat: 9050‐30‐0) was dissolved in 0.05 m MES buffer containing 25 mm EDC and 10 mm NHS to activate the carboxyl groups on the heparan sulfate. The scaffolds were then placed in the reaction solution and incubated at room temperature for 24 h with gentle shaking. This process allowed the negatively charged sulfonic groups (─SO_3_) on the heparan sulfate molecules to subsequently trap PDGF‐BB or PDGFB‐LG4 via electrostatic interaction. To immobilize PDGFB or PDGFB‐LG4, 5 µl of recombinant protein solution was injected into the scaffolds and incubated at 4 °C for 2 h.

### In Vitro Growth Factor Release Measurement

First, the PCLHS scaffold was loaded with 250 ng of growth factor and then transferred into 1.5 mL centrifuge tubes containing 200 µl of buffer (20 mm Tris−HCl, 150 mm NaCl, 0.1% BSA, pH 7.4). Every 24 h, the buffer was removed, stored at −80 °C, and replaced with fresh buffer. After 7 days, the cumulative release of the growth factor was quantified by ELISA (Proteintech, Cat: KE00161).

### In Vitro Osteogenesis Properties of HS, PDGFB, and PDGFB‐LG4

BMSCs were seeded on a twelve‐well plate (5 × 10⁴ cells per well). After 24 h, the medium was replaced with an osteogenic differentiation medium comprising 10 mm β‐glycerophosphate, 10 nm dexamethasone, and L‐ascorbic acid 2‐phosphate (50 µg mL^−1^). HS (500 ng mL^−1^), PDGFB (10 ng mL^−1^), and PDGFB‐LG4 (10 ng mL^−1^) were added to the osteogenic differentiation medium. The medium was replaced with fresh osteogenic differentiation medium every 3 days. After 7 days of culture, ALP staining was performed using the ALP staining kit (Beyotime, Cat: C3206). To quantitatively measure ALP activity, on days 7 and 14, the cells were washed twice with PBS and lysed using cell lysis buffer. After centrifugation at 10 000 rpm for 5 min, the supernatant from each sample was collected. ALP activity was measured using the Alkaline Phosphatase Assay Kit (Beyotime, Cat: P0321S) according to the manufacturer's instructions. The total protein content in the supernatant was determined using a BCA protein detection kit, and ALP activity was expressed relative to the total protein content. Alizarin red staining was performed after 14 days using the ARS staining kit (Beyotime, Cat: C0138). For the quantitative analysis of calcium deposition, 10% cetylpyridinium chloride was added to the stained cells. After incubation for 30 min at room temperature, the mixed solution was extracted. Then, 100 µL of the supernatant was transferred to a 96‐well plate, and the absorbance was measured at 562 nm using a microplate reader. The expression of typical osteogenic genes was detected by qRT‐PCR.

### Scratch Wound Healing Assay

BMSCs or HUVECs were seeded into 6‐well culture plates until they reached ≈95% confluence. A sterile 200 µL pipette tip was used to create a wound across each well, and the medium was replaced with a serum‐free medium containing 25 ng mL^−1^ of growth factor. At 0, 24, and 48 h, the cells were observed and photographed using an inverted microscope. The area of wound closure relative to the original wound area was measured using ImageJ software.

### Transwell Migration Assay

A 200 µL suspension of BMSCs (2 × 10⁴ cells/well) in serum‐free medium was seeded into the upper chamber. A total of 600 µL of serum‐free medium containing 30 ng mL^−1^ of growth factor was added to the lower chamber of 24‐well culture plates. After 18 h of incubation, the migrated cells were fixed with 4% paraformaldehyde for 30 min. Non‐migrated cells in the inserts were carefully removed with a cotton swab, and each insert was stained with 0.1% (w/v) crystal violet for 10 min. The cells on the lower side of the insert were photographed using an inverted microscope. The number of migrated cells was quantified using ImageJ software.

### Tube Formation Assay

Briefly, 50 µL of Matrigel (MedChemExpress, Cat: HY‐K6002) was added to 96‐well culture plates and placed in an incubator at 37 °C for 30 min. Once the Matrigel solidified, 2 × 10⁴ HUVECs were seeded onto the surface of each well and cultured with 30 ng mL^−1^ growth factor. After incubating at 37 °C for 4 h, the cells were observed under a light microscope to assess tube formation. Tubular‐network formation parameters were quantified in five random fields using ImageJ software.

### Cell Attachment Assay

First, 96‐well culture plates were coated with 4 µg of growth factor in 50 µl of D‐PBS overnight at 4 °C and then blocked for 60 min at room temperature with 200 µl of heat‐denatured Dulbecco's Modified Eagle's Medium (DMEM) containing 1% bovine serum albumin (BSA). The plates were washed twice with 0.1% blocking solution. BMSCs or HUVECs were washed twice, resuspended in 0.1% blocking solution, and used for the attachment assays. A total of 2 × 10⁴ cells/100 µl were added to each well and incubated for 3 h at 37 °C. Next, 10% CCK‐8 working solution was added to each well and incubated for 1 h. Finally, absorbance at 450 nm was measured using a microplate reader.

### Cell Proliferation Assay

First, BMSCs or HUVECs were seeded in a 96‐well cell culture plate (2000 cells per well) and incubated for 24 h. Then, the culture medium was replaced with DMEM containing 2% FBS and either 50 ng mL^−1^ HS, 30 ng mL^−1^ PDGFB, or 30 ng mL^−1^ PDGFB‐LG4. After 1, 3, and 5 days, the cell number was quantified using the CCK‐8 kit (Beyotime, Cat: C0037).

### Biocompatibility and Osteogenesis Evaluation of the Composite Scaffolds

To assess the biocompatibility of the composite scaffolds, 30 ng of PDGFB or PDGFB‐LG4 was loaded onto the PCLHS scaffold. First, 5000 BMSCs or HUVECs were seeded onto the scaffold in a 96‐well cell culture plate. After 1, 3, and 5 days, the cell number was quantified using the CCK‐8 kit. Cell adhesion and proliferation on the scaffolds were also analyzed using live‐dead staining (Beyotime, Cat: C2015S). On day 5, the samples were collected for live‐dead staining. To evaluate the osteogenesis of the composite scaffolds, 5 ng of PDGFB or PDGFB‐LG4 was loaded onto the PCLHS scaffold. First, 15 000 BMSCs were seeded onto the composite scaffolds and cultured in an osteogenic differentiation medium for 7 days. Then, the samples were collected for qPCR analysis.

### Critical‐Sized Calvarial Defect Model

All animal experiments were performed at Nanjing Jinling Hospital, Affiliated Hospital of Medical School, Nanjing University, China, with approval from the Animal Care and Use Committee, in accordance with international standards on animal welfare. A total of 36 male SD rats (250–300 g) were used to create bone defects (5 mm in diameter) on both sides of the calvarium. The rats were randomly divided into six groups: empty control (Control), PCL scaffolds (PCL), PCLHS scaffolds (PCLHS), PCLHS scaffolds with 1 µg PDGFB (PCLHS‐PDGFB1), PCLHS scaffolds with 5 µg PDGFB (PCLHS‐PDGFB5), and PCLHS scaffolds with 1 µg PDGFB‐LG4 (PCLHS‐PDGFB‐LG4). Briefly, the rats were shaved and anesthetized with 10% chloral hydrate (1 mL). A defect was created using a 5 mm trephine bur. The periosteum was completely removed, while the underlying dura mater remained intact. Three rats were sacrificed for each group at 4 and 8 weeks post‐surgery, resulting in six samples per group for analysis. The retrieved calvaria were fixed in 10% neutral buffered formalin for 48 h before further characterization.

### Micro‐CT Analysis

The harvested calvaria was examined using micro‐CT (Skyscan 1176, Bruker, Belgium) and analyzed with AVIZO software for 3D reconstruction and image visualization. A cylindrical region of interest with a 5 mm diameter, centered on the calvarial defect area, was selected for analysis. Bone volume to total volume (BV/TV) and defect coverage were quantified using AVIZO software (Thermo Scientific, USA).

### Histological and Immunohistochemistry Analysis

After micro‐CT analysis, the samples were decalcified by soaking in a decalcifying solution for one month at 37 °C. The samples were then dehydrated in a gradient series of alcohol, embedded in paraffin, and sectioned into 5 µm slices. The slices were stained with hematoxylin and eosin (HE) and Masson's trichrome. To further assess angiogenesis and new bone formation following scaffold implantation, immunofluorescence staining for CD31 (Abcam Cat: ab28364), COL1 (Abcam Cat: ab308221), and RUNX2 (Abcam Cat: 192256) was performed at 8 weeks. The fluorescence signal intensity was analyzed using ImageJ software.

### Statistics Analysis

All in vitro analyses were performed in triplicate, and the in vivo analyses were conducted with six independent experiments. All data are expressed as the mean ± standard deviation. Statistical analysis was performed using one‐way or two‐way analysis of variance (ANOVA) with Tukey's multiple comparisons test, using GraphPad Prism 9.5 software. A *p*‐value of less than 0.05 was considered statistically significant: ^*^ *p* < 0.05, ^**^ *p* < 0.002, and ^***^ *p* < 0.001.

## Conflict of Interest

The authors declare no conflict of interest.

## Supporting information



Supporting Information

## Data Availability

The data that support the findings of this study are available from the corresponding author upon reasonable request.

## References

[1] G. L. Koons , M. Diba , A. G. Mikos , Nat. Rev. Mater. 2020, 5, 584.

[2] H. Xie , Z. Cui , L. Wang , Z. Xia , Y. Hu , L. Xian , C. Li , L. Xie , J. Crane , M. Wan , G. Zhen , Q. Bian , B. Yu , W. Chang , T. Qiu , M. Pickarski , L. T. Duong , J. J. Windle , X. Luo , E. Liao , X. Cao , Nat. Med. 2014, 20, 1270. 25282358 10.1038/nm.3668 PMC4224644

[3] P. Carmeliet , R. K. Jain , Nature 2011, 473, 298. 21593862 10.1038/nature10144 PMC4049445

[4] P. S. Briquez , L. E. Clegg , M. M. Martino , F. M. Gabhann , J. A. Hubbell , Nat. Rev. Mater. 2016, 1, 15006.

[5] M. H. Hettiaratchi , L. Krishnan , T. Rouse , C. Chou , T. C. McDevitt , R. E. Guldberg , Sci. Adv. 2020, 6, 1240. 10.1126/sciadv.aay1240 PMC6941907 31922007

[6] C. L. Hastings , E. T. Roche , E. Ruiz‐Hernandez , K. Schenke‐Layland , C. J. Walsh , G. P. Duffy , Adv. Drug Delivery Rev. 2015, 84, 85. 10.1016/j.addr.2014.08.006 25172834

[7] M. M. Martino , P. S. Briquez , E. Güç , F. Tortelli , W. W. Kilarski , S. Metzger , J. J. Rice , G. A. Kuhn , R. Müller , M. A. Swartz , J. A. Hubbell , Science 2014, 343, 885. 24558160 10.1126/science.1247663

[8] J. Lee , J. J. Yoo , A. Atala , S. J. Lee , Biomaterials 2012, 33, 6709. 22770570 10.1016/j.biomaterials.2012.06.017 PMC3760265

[9] S. Murali , B. Rai , C. Dombrowski , J. L. Lee , Z. X. Lim , D. S. Bramono , L. Ling , T. Bell , S. Hinkley , S. S. Nathan , J. H. Hui , H. K. Wong , V. Nurcombe , S. M. Cool , Biomaterials 2013, 34, 5594. 23632323 10.1016/j.biomaterials.2013.04.017

[10] C. Dombrowski , S. J. Song , P. Chuan , X. Lim , E. Susanto , A. A. Sawyer , M. A. Woodruff , D. W. Hutmacher , V. Nurcombe , S. M. Cool , Stem Cells Dev. 2008, 18, 661. 10.1089/scd.2008.0157 18690792

[11] K. Hozumi , N. Suzuki , P. K. Nielsen , M. Nomizu , Y. Yamada , J. Biol. Chem. 2006, 281, 32929. 16945929 10.1074/jbc.M605708200

[12] M. Mochizuki , E. Güç , A. J. Park , Z. Julier , P. S. Briquez , G. A. Kuhn , R. Müller , M. A. Swartz , J. A. Hubbell , M. M. Martino , Nat. Biomed. Eng. 2020, 4, 463. 31685999 10.1038/s41551-019-0469-1

[13] Y. Niu , Q. Li , Y. Ding , L. Dong , C. Wang , Adv. Drug Deliv. Rev. 2019, 146, 190. 29879493 10.1016/j.addr.2018.06.002

[14] A. S. Lee , M. Inayathullah , M. A. Lijkwan , X. Zhao , W. Sun , S. Park , W. X. Hong , M. B. Parekh , A. V. Malkovskiy , E. Lau , X. Qin , V. R. Pothineni , V. Sanchez‐Freire , W. Y. Zhang , N. G. Kooreman , A. D. Ebert , C. K. F. Chan , P. K. Nguyen , J. Rajadas , J. C. Wu , Nat. Biomed. Eng. 2018, 2, 104. 29721363 10.1038/s41551-018-0191-4 PMC5927627

[15] M. M. Nguyen , A. S. Carlini , M. P. Chien , S. Sonnenberg , C. Luo , R. L. Braden , K. G. Osborn , Y. Li , N. C. Gianneschi , K. L. Christman , Adv. Mater. 2015, 27, 5547. 26305446 10.1002/adma.201502003 PMC4699559

[16] S. Huang , D. Lei , Q. Yang , Y. Yang , C. Jiang , H. Shi , B. Qian , Q. Long , W. Chen , Y. Chen , L. Zhu , W. Yang , L. Wang , W. Hai , Q. Zhao , Z. You , X. Ye , Nat. Med. 2021, 27, 480. 33723455 10.1038/s41591-021-01279-9

[17] J. Duan , D. Lei , C. Ling , Y. Wang , Z. Cao , M. Zhang , H. Zhang , Z. You , Q. Yao , Regen Biomater. 2022, 9, rbac033. 35719204 10.1093/rb/rbac033 PMC9201971

[18] D. Lei , Y. Yang , Z. Liu , B. Yang , W. Gong , S. Chen , S. Wang , L. Sun , B. Song , H. Xuan , X. Mo , B. Sun , S. Li , Q. Yang , S. Huang , S. Chen , Y. Ma , W. Liu , C. He , B. Zhu , E. M. Jeffries , F.‐L. Qing , X. Ye , Q. Zhao , Z. You , Mater. Horiz. 2019, 6, 1197.

[19] Y. Ha , X. Ma , S. Li , T. Li , Z. Li , Y. Qian , M. Shafiq , J. Wang , X. Zhou , C. He , Adv. Funct. Mater. 2022, 32, 2200011.

[20] B. Grigoryan , S. J. Paulsen , D. C. Corbett , D. W. Sazer , C. L. Fortin , A. J. Zaita , P. T. Greenfield , N. J. Calafat , J. P. Gounley , A. H. Ta , F. Johansson , A. Randles , J. E. Rosenkrantz , J. D. Louis‐Rosenberg , P. A. Galie , K. R. Stevens , J. S. Miller , Science 2019, 364, 458. 31048486 10.1126/science.aav9750 PMC7769170

[21] G. Matthew , Y. Yao , V. G. William , W. Hom‐Lay , Plastic and Aesthetic Research 2021, 8, 3.35765666

[22] J. W. Hustedt , D. J. Blizzard , Yale J. Biol. Med. 2014, 87, 549. 25506287 PMC4257039

[23] S. Laner‐Plamberger , M. Oeller , E. Rohde , K. Schallmoser , D. Strunk , Int. J. Mol. Sci. 2021, 22, 5178. 34769471 10.3390/ijms222112041 PMC8584295

[24] R. A. Jackson , S. Murali , A. J. van Wijnen , G. S. Stein , V. Nurcombe , S. M. Cool , J. Cell. Physiol. 2007, 210, 38. 17051597 10.1002/jcp.20813

[25] C. Dombrowski , S. J. Song , P. Chuan , X. Lim , E. Susanto , A. A. Sawyer , M. A. Woodruff , D. W. Hutmacher , V. Nurcombe , S. M. Cool , Stem Cells Dev. 2009, 18, 661. 18690792 10.1089/scd.2008.0157

[26] L. Yu , H. Qu , Y. Yu , W. Li , Y. Zhao , G. Qiu , J. Cell. Mol. Med. 2018, 22, 6134. 30338912 10.1111/jcmm.13892 PMC6237555

[27] M. M. Martino , P. S. Briquez , K. Maruyama , J. A. Hubbell , Adv. Drug Delivery Rev. 2015, 94, 41. 10.1016/j.addr.2015.04.007 25895621

[28] N. Laurel , P. W. Jack , Plastic and Aesthe. Res. 2019, 6, 21.

[29] A. I. Caplan , D. Correa , J. Ortho. Res. 2011, 29, 1795. 10.1002/jor.21462 21618276

[30] E. Friedlaender Gary , S. Lin , A. Solchaga Luis , B. Snel Leo , E. Lynch Samuel , Curr. Pharm. Des. 2013, 19, 3384. 23432673 10.2174/1381612811319190005

[31] M. Kaipel , S. Schützenberger , A. Schultz , J. Ferguson , P. Slezak , T. J. Morton , M. Van Griensven , H. Redl , J. Ortho. Res. 2012, 30, 1563. 10.1002/jor.22132 22508566

[32] P. Simon‐Assmann , G. Orend , E. Mammadova‐Bach , C. Spenlé , O. Lefebvre , Int. J. Dev. Biol. 2011, 55, 455. 21858771 10.1387/ijdb.103223ps

[33] C. S. Barros , S. J. Franco , U. Müller , Cold Spring Harb. Perspect. Biol. 2011, 3, a005108. 21123393 10.1101/cshperspect.a005108 PMC3003458

[34] V. Iorio , L. D. Troughton , K. J. Hamill , Adv. Wound Care 2015, 4, 250. 10.1089/wound.2014.0533 PMC4397997 25945287

[35] G. Damodaran , W. H. C. Tiong , R. Collighan , M. Griffin , H. Navsaria , A. Pandit , J. Biomed. Mater. Res., Part A 2013, 101, 2788. 10.1002/jbm.a.34583 23463686

[36] A. Domogatskaya , S. Rodin , K. Tryggvason , Annu. Rev. Cell Dev. Biol. 2012, 28, 523. 23057746 10.1146/annurev-cellbio-101011-155750

[37] I. Senyürek , W. E. Kempf , G. Klein , A. Maurer , H. Kalbacher , L. Schäfer , I. Wanke , C. Christ , S. Stevanovic , M. Schaller , P. Rousselle , C. Garbe , T. Biedermann , B. Schittek , J. Innate. Immun. 2014, 6, 467. 24458132 10.1159/000357032 PMC6741626

[38] R. O. Sigle , S. G. Gil , M. Bhattacharya , M. C. Ryan , T.‐M. Yang , T. A. Brown , A. Boutaud , Y. Miyashita , J. Olerud , W. G. Carter , J. Cell Sci. 2004, 117, 4481. 15316072 10.1242/jcs.01310

[39] L. Mao , L. Wang , J. Xu , J. Zou , Cell Death Discov. 2023, 9, 119. 37037822 10.1038/s41420-023-01417-x PMC10086008

[40] Z. Hamidouche , O. Fromigué , J. Ringe , T. Häupl , P. Vaudin , J. C. Pagès , S. Srouji , E. Livne , P. J. Marie , Proc. Natl. Acad. Sci. USA 2009, 106, 18587. 19843692 10.1073/pnas.0812334106 PMC2773973

[41] R. Patricia , C. Sonia , C. Hanane , D. Guila , P. Didier , H. Benjamin , Eur. J. Dermatol. 2013, 10.1684/ejd.2013.1974.

[42] H. A. Multhaupt , A. Yoneda , J. R. Whiteford , E. S. Oh , W. Lee , J. R. Couchman , J. Physiol. Pharmacol. 2009, 60, 31. 20083849

[43] A. Ho‐Shui‐Ling , J. Bolander , L. E. Rustom , A. W. Johnson , F. P. Luyten , C. Picart , Biomaterials 2018, 180, 143. 30036727 10.1016/j.biomaterials.2018.07.017 PMC6710094

[44] L. Chou , Y. Chang , K. Lan , M. Liu , Y. Lu , X. Li , P. Li , Y. Xu , J. Int. Med. Res. 2022, 50, 03000605221141312. 36495169 10.1177/03000605221141312 PMC9747886

[45] Q. Gao , Y. Hou , Z. Li , J. Hu , D. Huo , H. Zheng , J. Zhang , X. Yao , R. Gao , X. Wu , L. Sui , J. Cell. Mol. Med. 2021, 25, 6695. 34114337 10.1111/jcmm.16672 PMC8278073

[46] G. L. Saux , M. C. Wu , E. Toledo , Y. Q. Chen , Y. J. Fan , J. C. Kuo , M. Schvartzman , ACS Appl. Mater. Interfaces 2020, 12, 22399. 32323968 10.1021/acsami.9b20939

[47] P. S. Briquez , H.‐M. Tsai , E. A. Watkins , J. A. Hubbell , Sci. Adv. 7, eabh4302. 34117071 10.1126/sciadv.abh4302 PMC8195475

[48] O. Tsigkou , I. Pomerantseva , J. A. Spencer , P. A. Redondo , A. R. Hart , E. O'Doherty , Y. Lin , C. C. Friedrich , L. Daheron , C. P. Lin , C. A. Sundback , J. P. Vacanti , C. Neville , Proc. Natl. Acad. Sci. USA 2010, 107, 3311. 20133604 10.1073/pnas.0905445107 PMC2840421

[49] C. Fields , A. Cassano , C. Allen , A. Meyer , K. J. Pawlowski , G. L. Bowlin , S. E. Rittgers , M. Szycher , J. Biomater. Appl. 2002, 17, 45. 12222757 10.1177/0885328202017001861

[50] M. G. Tonnesen , X. Feng , R. A. F. Clark , J. Investig. Dermatol. Symp. Proc. 2000, 5, 40. 10.1046/j.1087-0024.2000.00014.x 11147674

[51] M. Crisan , S. Yap , L. Casteilla , C.‐W. Chen , M. Corselli , T. S. Park , G. Andriolo , B. Sun , B. Zheng , L. Zhang , C. Norotte , P.‐N. Teng , J. Traas , R. Schugar , B. M. Deasy , S. Badylak , H.‐J. Bűhring , J.‐P. Giacobino , L. Lazzari , J. Huard , B. Péault , Cell Stem Cell 2008, 3, 301. 18786417 10.1016/j.stem.2008.07.003

[52] S. Mannsfeld , C. K. Tee , R. M. Stoltenberg , H. H. Chen , S. Barman , B. Muir , A. N. Sokolov , C. Reese , Z. Bao , Nat. Mater. 2010, 9, 859. 20835231 10.1038/nmat2834

[53] N. Ferrara , H. P. Gerber , J. J. N. M. Lecouter , Nat. Med. 2003, 9, 669. 12778165 10.1038/nm0603-669

[54] P. Brooks , R. Clark , D. J. S. Cheresh , 1994, Science 264, 569. 7512751 10.1126/science.7512751

[55] B. P. Eliceiri , D. A. Cheresh , Curr. Opin. Cell Biol. 2001, 13, 563. 11544024 10.1016/s0955-0674(00)00252-0

