# Supplementary material for: 3D‐Printed Biomimetic Vascular Scaffold Crosslinked with Heparan Sulfate for Sustained Release of PDGFB‐LG4 Fusion Protein Promotes Bone Regeneration
Source: Adv Sci (Weinh). 2025 Mar 27;12(21):2414362. doi: 10.1002/advs.202414362 (PMC12140322; doi:10.1002/advs.202414362)
Supplement: Supplementary file 1 — Supporting Information [file ADVS-12-2414362-s001.docx]

**Supplement information**

**3D-Printed Biomimetic Vascular Scaffold Crosslinked with Heparan Sulfate for Sustained Release of PDGFB-LG4 Fusion Protein Promotes Bone Regeneration**

| Recombinant protein | Amino acid sequence |
| --- | --- |
| PDGFB | MNRCWALFLSLCCYLRLVSAHHHHHHEGDPIPEELYEMLSDHSIRSFDDLQRLLHGDPGEEDGAELDLNMTRSHSGGELESLARGRRSLGSLTIAEPAMIAECKTRTEVFEISRRLIDRTNANFLVWPPCVEVQRCSGCCNNRNVQCRPTQVQLRPVQVRKIEIVRKKPIFKKATVTLEDHLACKCETVAAARPVT |
| PDGFB-LG4 | MNRCWALFLSLCCYLRLVSAEGDPIPEELYEMLSDHSIRSFDDLQRLLHGDPGEEDGAELDLNMTRSHSGGELESLARGRRSLGSLTIAEPAMIAECKTRTEVFEISRRLIDRTNANFLVWPPCVEVQRCSGCCNNRNVQCRPTQVQLRPVQVRKIEIVRKKPIFKKATVTLEDHLACKCETVAAARPVTGGGSGGGSYVAGAHQFGLSQNSHLVLPLNQSDVRKRLQVQLSIRTFASSGLIYYVAHQNQMDYATLQLQEGRLHFMFDLGKGRTKVSHPALLSDGKWHTVKTEYIKRKAFMTVDGQESPSVTVVGNATTLDVERKLYLGGLPSHYRARNIGTITHSIPACIGEIMVNGQQLDKDRPLSASAVDRCY |

Table S1 Recombinant protein amino acid sequence


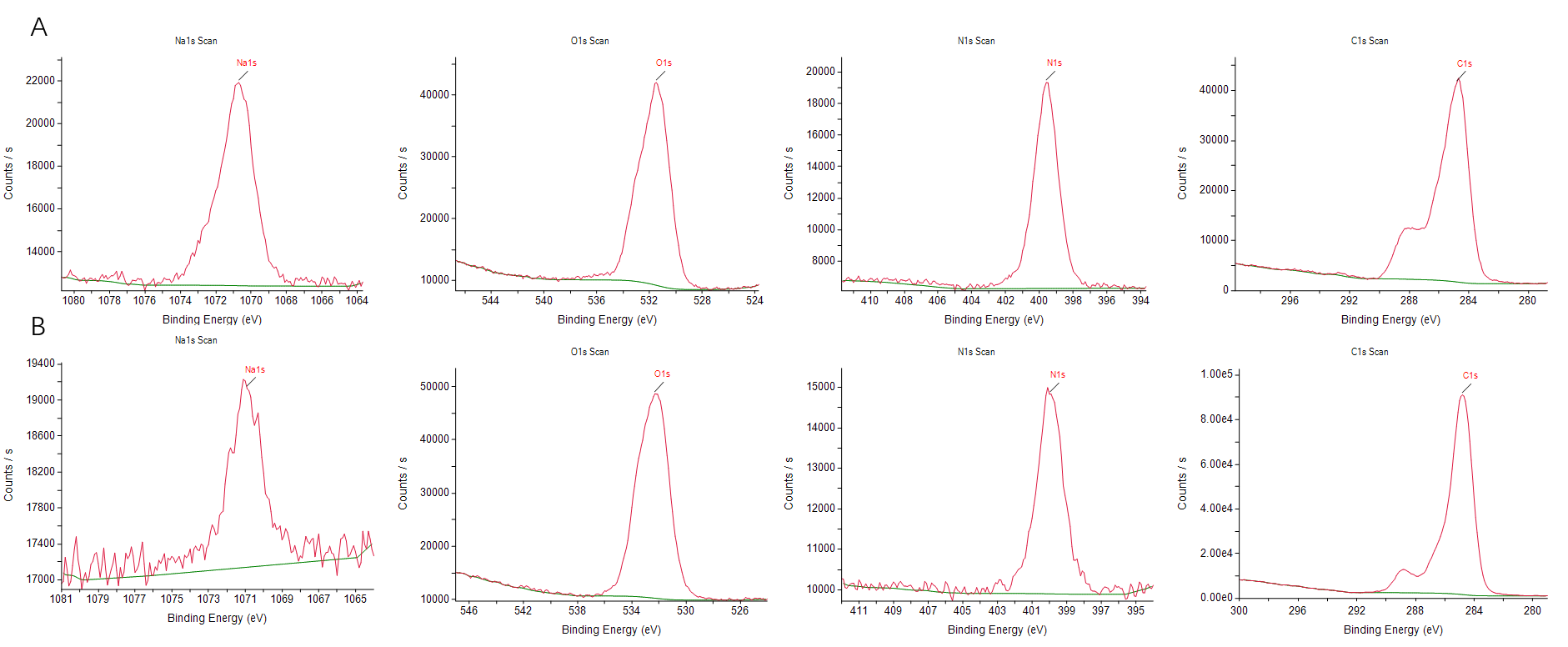


**Figure S1.** XPS results for Na, O, N, and Cl elements in PCL and PCLHS scaffolds. A) PCL scaffold. B) PCLHS scaffold.


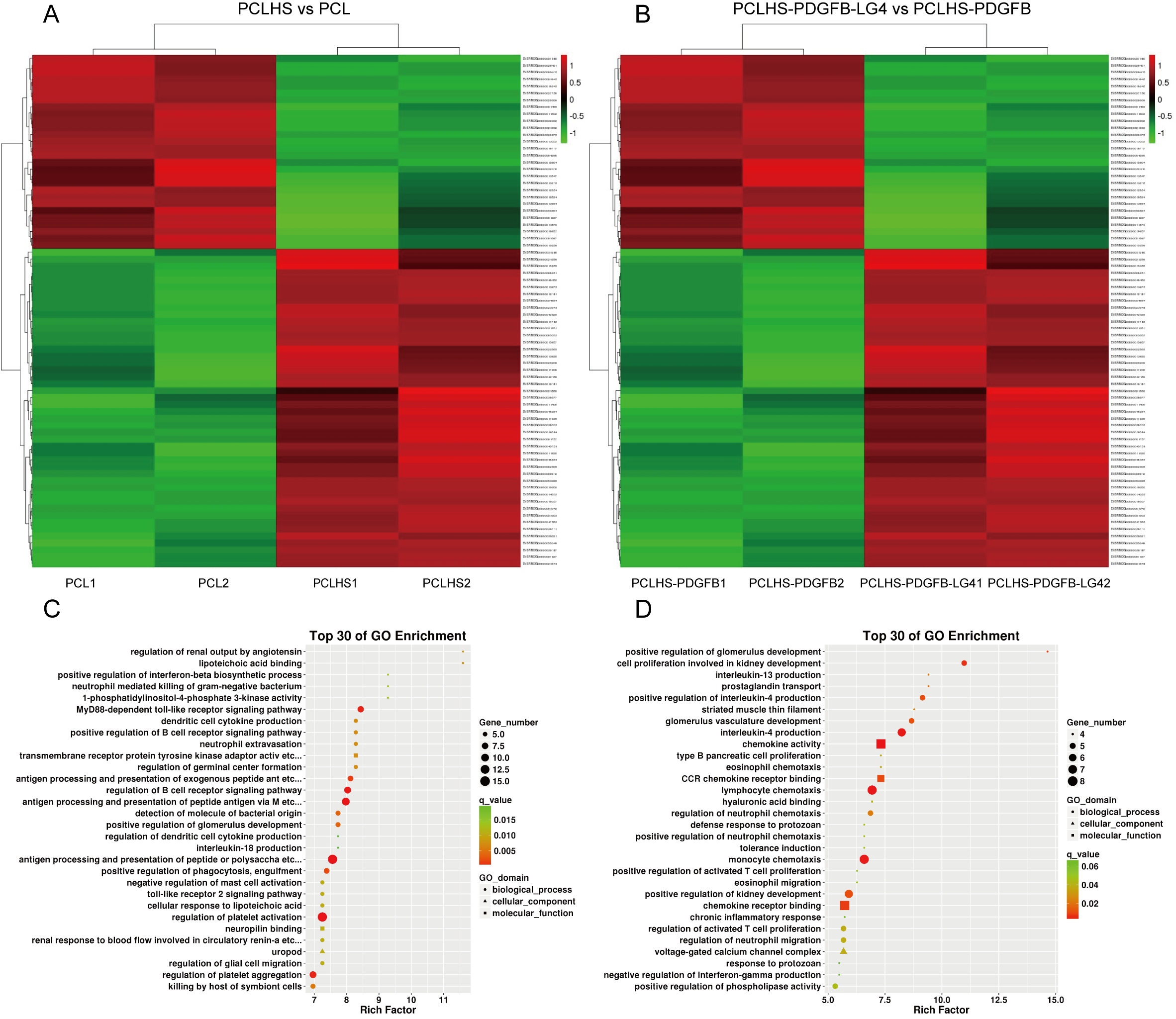


**Figure S2.** RNA-seq results of the composite scaffolds. A, C) Comparison of heatmap and GO enrichment analysis between the PCLHS scaffold and PCL scaffold. B, D) Comparison of heatmap and GO enrichment analysis between the PCLHS-PDGFB-LG4 scaffold and PCLHS-PDGFB scaffold.


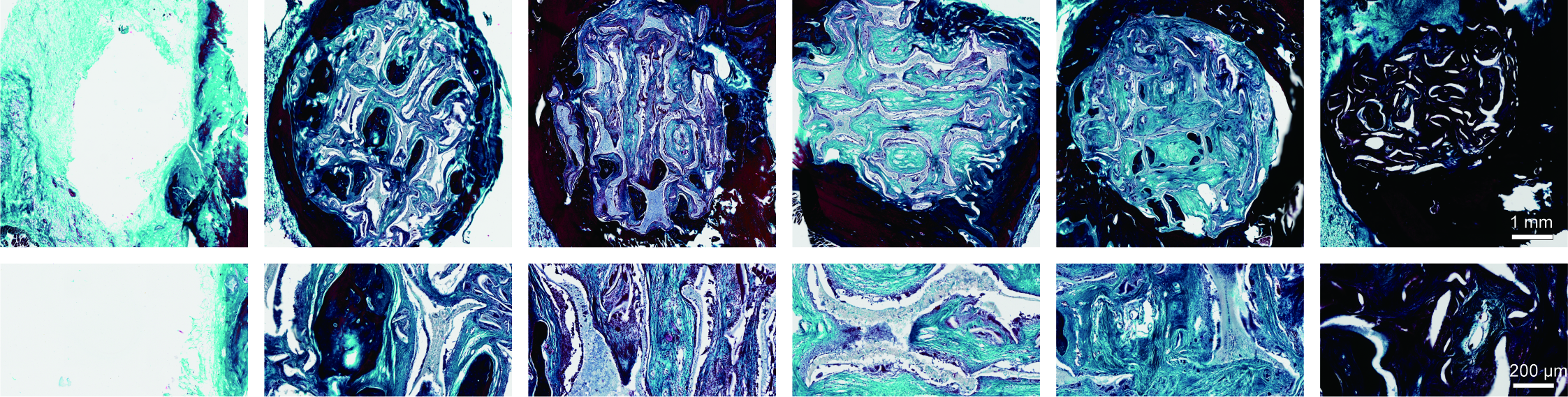


**Figure S3.** Masson’s staining of the top view of bone defects in rats at 8 weeks.
